# Effect of Green Algae *Chaetomorpha antennina* Extract on Growth, Modulate Immunity, and Defenses against *Edwardsiella tarda* Infection in *Labeo rohita*

**DOI:** 10.3390/ani10112033

**Published:** 2020-11-04

**Authors:** Govindharajan Sattanathan, Vairakannu Tamizhazhagan, Swaminathan Padmapriya, Wen-Chao Liu, Balamuralikrishnan Balasubramanian

**Affiliations:** 1 Department of Life Science, MASS College of Arts and Science, Kumbakonam 612 501, Tamil Nadu, India; sattanathanphd@gmail.com; 2 Department of Zoology, Syed Ammal Arts and Science College, Ramanathapuram 623 513, Tamil Nadu, India; tamilzoon@gmail.com; 3 Department of Zoology, Dharmapuram Gnanambigai Government Arts College for Women, Mayiladuthurai 609 001, Tamil Nadu, India; spsrijan23@gmail.com; 4 Department of Animal Science, College of Coastal Agriculture Science, Guangdong Ocean University, Zhanjiang 524088, China; 5 Department of Food Science and Biotechnology, College of Life Science, Sejong University, Seoul 05006, Korea

**Keywords:** MECA, growth performance, immunostimulant, rohu, challenge test

## Abstract

**Simple Summary:**

Global demand for macroalgal and microalgal foods is growing, and algae are increasingly being consumed for functional benefits beyond the traditional considerations of nutrition and health. The study was undertaken to know the effect of methanolic extract of *Chaetomorpha antennina* in fish. The results demonstrated that the betterment of growth, immune system, and resistance to disease against *Edwardsiella tarda* in Indian major carp, rohu, and *Labeo rohita*. These findings are useful for development of new feed additive in aquaculture sectors.

**Abstract:**

The current study focused on assessing the outcome of methanol extract of *Chaetomorpha antennina* (MECA) on the growth performance and immune modulation in both specific and non-specific immune responses through the assessment of neutrophil, serum lysozyme, serum myeloperoxidase, antiprotease, ceruloplasmin, reactive oxygen species (ROS), and reactive nitrogen species (RNS) activity in *Labeo rohita* (rohu) at 28 days post treatment along with assessment of the disease resistance capacity against *Edwardsiella tarda* at 30days post immunization. Fishes (*n* = 144; average weight 50.0 ± 0.23 g) were evenly divided into four treatments, with 12 fishes per tank in triplicates. The MECA was injected intraperitoneally in the fishes at different doses as 0, 25, 75, and 150 mg/kg of the body weight. The results demonstrated that fish treated with MECA have an increased body weight, specific growth rate, and feed conversion ratio (*p* < 0.05) with respect to the control group. Results suggested that the MECA inclusion can significantly enhance (*p* < 0.05) the levels of serum lysozyme, neutrophil function, serum antiprotease activity, cellular RNS, and ROS production. Exposure to MECA of 75 mg/kg showed a significantly higher survival percentage against *E. tarda* disease infection. These results indicate MECA as a stimulant of immunity in *L. rohita* against *E. tarda*. The results suggested that MECA is a potent immunostimulant in finfish aquaculture and can offer higher economic welfare.

## 1. Introduction

The aquaculture sector is sparkling over the years due to strongly integrating with developing food safety, dietetic desires of the world populace, and providing employment as a spawn for foreign trade. Transversely, diversification and intensification practices in aquaculture instigation havebeen elevated with the raise of transmittable disease indices. Thus, recent researchers havemainly focused on developing resistance mechanisms in fishes against several microbial attacks using natural components as it is a prerequisite to identify the mechanism behind fish disease due to developing fiscal significance in aquaculture [1]. One of the most common bacterial diseases in fish aquaculture is Edwardsiellosis caused by *Edwardsiella tarda*. A wide range of freshwater and marine fish are infected by this pathogen apart from a variety of different animals, including reptiles, amphibians, birds, and humans [2,3]. The Channel catfish (*Ictalurus punctatus*), African catfish (*Clariasgariepinus*), striped bass (*Moronesaxatilis*), European eel (*Anguilla anguilla*) and Japanese eel (*Anguilla japonica*), olive flounder (*Paralichthysolivaceus*) and turbot (*Psettamaximus*), and Indian major carps (*Labeorohita*, *Cirrhinusmrigala*, and *Catlacatla*) are economically important cultivated fish species affected by Edwardsiellosis in different geographical areas [4]. Available immuno-stimulants to tackle with the fish disease have connotation as they provide an alternative to others that lack competence against most of the drugs and antibiotics with exorbitant costs [5,6]. In certain countries, herbs are used to boost the naïve resistance within the body to enhance immune substance production against various diseases [5]. When compared to chemotherapeutic agents, herbal stuffs are a cheaper source for therapeutics and have a wider range of accuracy and feasibility for all problems in aquaculture [6,7]. Algae-derived natural metabolites can be extensively utilized in the pharmacognosy field and medical chemistry [7]. Especially, marine algae is a rich source of potentially bioactive components with positive effects in improving one’s health by developing the body’s natural disease resistance capability [8]. The *Chaetomorpha* sp. has been recognized as a good additive to promote *Penaeus monodon* growth performance and feed efficiency [9,10]. The extracts of *Chaetomorpha antennina* have been studied and discovered to have antibacterial, antifungal, anti-inflammatory, antimalarial, antioxidant, antidote, and radical scavenging properties. They are also seen to be effective against hypertension and tumors [11]. In streptozotocin-induced rats, the use of *C. antennina* with methanolic extract showed antidiabetic activity [12,13,14,15]. A dietary combination of *C. linum* and *Zostera marina*, *Apostichopus japonicus* was found to influence the food utilization, energy levels, and development in sea cucumber [16,17]. Studies on brown, red, and green algae components illustrated reduced oxidation and increased antimicrobial, antitumoral, anticoagulant, antivirals, antifouling, antihelminthic, and antifungal activities [18,19,20,21]. Ours is a novel study with theaim to explore the effect of MECA algae on development of specific and non-specific immunity along with disease resistance capacity in *Labeo rohita*. 

## 2. Materials and Methods

### 2.1. Fish and Experimental Setup

The experimental protocol for this study was reviewed and approved by the Animal Care and Use Institutional Committee (No. 20190125). Healthy *L. rohita* (initial mean weight 50.0 ± 0.23 g) were bought from the local fish farm (Kumbakonam, Tamil Nadu, India) for the trial. Before pioneering the experiment, the fishes were acclimatized with the experimental conditions for nearly two weeks in plastic tanks (150 L) reinforced with fiber. The basic physico-chemical parameters of water were measured systematically throughout the period of the experiment to maintain the optimum level (dissolved oxygen: 6.25 ± 0.24 mg L^−1^; pH: 8.01 ± 0.23; ammonia: 0.024 ± 0.021 mg L^−1^; nitrite: 0.019 ± 0.022 mg L^−1^). The water temperature during the experiment was 28–30 °C. The basal diet was formulated to supply 35% crude protein and 3000 kcal digestible energy/kg diet as stated by NRC [22]. Daily water exchange was carried out for proper removal of the waste feed and fecal material. 

### 2.2. Preparation of Basal Diet

The basal ingredients included rice bran, groundnut oil cake, fish meal, soybean meal, and vitamin and mineral mix. First, dry ingredients were mixed thoroughly with 1% starch addition. Water was added and mixed thoroughly in a mixer for 20 min. The resulting dough was pelleted and dried at room temperature for 48 h followed by storage in airtight containers at room temperature until fed [23]. Fishes were fed abasal diet at a rate of 4% based on their body weight per day throughout the experiment. The daily ration was subdivided in two and wasfed at 9.00 h and 17.00 h. According to AOAC [24] criterion, the diets were analyzed to check the proximate relationship. The criteria includes the estimation of moisture content after being dried at 105 °C in the hot air oven until aconstant weight was obtained, and the usage of the Kjeldahl method after acid digestion to estimate the level of crude protein (N × 6.25) and to determine the ash content by incinerating them at 500 °C in a muffle furnace for about 18 h. The approximate constitution of the basal diet consists of crude proteins of 34.5%, lipids with 7.1%, ash of 15.3%, moisture of 6.9%, and fiber of 2.8%. 

### 2.3. Preparation of MECA

During the low-tide period, *C. antennina* was collected from Parangipettai, Chidamparam, Cuddalore District, Tamil Nadu, India. The algal specimens were thoroughly washed with running tap water and left to dry at room temperature for 4–7 days. Approximately 1.0 cm of all the parts of the algae were cut into small pieces and used for extraction. The extraction was carried out as described by Kriubakaran et al. [25]. For 3 days at room temperature (30 ± 2 °C), 100 g of small pieces of algae were soaked in 1000 mL of methanol. To ensure homogeneity during the process of extraction, the mixture was stirred and filtered by filtered paper (Whatman No. 1, HiMedia, Bengaluru, India). In order to obtain a dark oily paste of concentrated extract, the filtrate was collected and evaporated. Sterile distilled water was used to reconstitute the methanolic extract. 

### 2.4. Experimental Design

*L. rohita* fishes (*n* = 144) were evenly distributed into 12 tanks (12 fish per tank) in triplicate. MECA reconstituted with sterile distilled water was given through intraperitoneal injection with 0.2 mL of MECA for fishes with increasing dose levels at 0, 25, 75, and 150 mg/kg of the total body weight (control, T1, T2, and T3, respectively) to investigate the specific and nonspecific immune responses. In total, 0.2 mL of sterile water wereincluded in the control group. Bleeding of the fishes was observed at several durations prior to 7 daysand 7, 14, 21, and 28 days post treatment. The weight of each fish in the experimental group was recorded individually for sampling individually and estimating the total biomass in the tank. These data were used to calculate the feed conversion ratio (FCR) and specific growth rate (SGR). 

### 2.5. Blood Collection and Sample Analysis for Immunological Parameters

At 0, 7, 14, 21, and 28 days, 6 fish from each tank were randomly captured for blood collection. Blood was withdrawn from caudal venipuncture from each fish sample in a 2-mL sterilized tuberculin 24-gauge needle. One of each blood sample was transferred to a microtube containing heparin anticoagulant and immediately used for assays while the other half was transferred to a non-heparinized microtube, placed at room temperature, and allowed to clot for 2 h. Serum was separatedby centrifugation at 1500× *g* for 20 min and stored at −20 °C until use [26]. 

#### 2.5.1. Neutrophil Activity

The respiratory burst activity of the neutrophils was measured by the Nitro Blue tetrazolium (NBT) assay following the method stated by Stasiak and Bauman [27]. Blood (100 µL) was placed in the wells of aflat-bottom micro titer plate and incubated at 37 °C for 1 h to allow adhesion of cells. Thesupernatant was discarded and the wells were washed three times with Phosphate-buffered saline (PBS). After washing, 100 µL of 0.2% NBT were added and incubated for 1 h. The cells were then fixed with 100% methanol for 2–3 min and washed three times with 70% methanol. The plates were air-dried and 120 µL of 2 N potassium hydroxide and 140 µL dimethyl sulphoxide were added to each well. The Optical Density (OD) was recorded by an ELISA (BioTek Power Wave 340, Mumbai, India) reader at 620 nm. 

#### 2.5.2. Serum Lysozyme Activity

Lysozyme activity was measured by the method of Parry et al. [28] with minor modifications done by Hutchinson and Manning [29]. In this turbidimetric assay, 0.03% lyophilized *Micrococcus luteus* in 0.05 mM sodium phosphate buffer (pH 6.2) was used as substrate. About 10 µL of fish serum wereadded to 250 µL of bacterial suspension in a duplicate well with ‘a U’ bottom microtitre plate and the reduction in absorbance at 490 nm was determined after 0.5 and 4.5 min of incubation at 22 °C using a microplate reader. One unit of lysozyme activity was defined as the reduction in absorbance at 0.001 per min. 

#### 2.5.3. Myeloperoxidase (MPO) Activity

The total MPO content present in serum was measured according to Quade and Roth [30] with slight modification [31]. About 15 µL of serum were diluted with 135 µL of Hank’s Balanced Salt Solution (HBSS) without Ca2+ or Mg2+ in 96-well plates. Then, 25 µL of 20 mM 3,3′-5,5′tetramethylbenzidine hydrochloride (TMB) (HiMedia, Bengaluru, India) and 25 µL of 5 mM H_2_O_2_ (Qualigens, Mumbai, India) (both substrates of MPO and prepared on the same day) were added. The color change reaction was stopped after 2 min by adding 50 mL of 4 M sulphuric acid (H_2_SO_4_). The plate was centrifuged at 400× *g* for 10 min, and 150 mL of the supernatants, present in each well, were transferred to new 96-well plates. The OD was recorded at 450 nm in a microplate reader. 

#### 2.5.4. Ceruloplasmin Activity

The method of p-phenylene diamine (PPD) oxidase activity had minor modifications and was used to measure the activity of ceruloplasmin [32]. The whole serum was added to acetate buffer with pH 5.0 and 1.2 M concentration, to a volume of 0.5 µL, with the substrate of 0.1% PPD. Then, 0.5 mL of 0.5% sodium azide (NaN_3_) were added to the serum of 25 µL to prepare the blank. The incubation of both solutions wasperformed at 30 °C for half an hour. Then, 0.5 µL of 0.5% NaN3 wereadded to stop the reaction. The absorbance reduction of about 0.001 per min at the wavelength of 550 nm was catalyzed with a certain amount of oxidase seen and wasdefined as the one unit of ceruloplasmin. 

#### 2.5.5. Serum Antiprotease Activity

The total antiprotease activity was determined according to Rao and Chakrabarti [33] with minor modifications. About 10 µL of undiluted serum wereinitially incubated with trypsin solution in duplicates (Trypsin bovine pancreas, in 0.01 M TrisHCl, pH 8.2, HiMedia, Bengaluru, India), followed byaddition of 500 µL of 2 mM BAPNA (sodium-benzoyl-DL-arginine-p-nitroanilide HCL, HiMedia, Bengaluru, India) substrate to make up the volume to 1 mL with 0.1 M Tris HCL (pH 8.2) with incubation at 22 °C for 25 min. The reaction was stopped with 30% acetic acid and optical density was read at 415 nm in a microplate reader against the blank. The inhibitory capacity of antiprotease was expressed in terms of the percentage of trypsin inhibition as described by Rao and Chakrabarthi [33]:

Percent inhibition (%) = (OD reference × OD sample/OD reference) × 100.(1)

### 2.6. Preparation of Viable Leukocytes from Peripheral Blood

The fishes were subjected to bleeding by using a 5–10-mL syringe that was packed with RPMI-1640 medium supplemented with 50,000 IU^−1^ sodium heparin, 100,000 IU^−1^ penicillin, and 100 mg L^−1^ streptomycin as medium to collect the blood and to separate leukocytes from the peripheral blood. Then, an equal volume of medium was used to separate the lymphocytes (Lymphosep, ICN Biomedicals, Inc., Irvine, CA, USA) to overlay from the diluted blood. Centrifugation was done at 800× *g* for 20 min. The interface leukocytes were collected followed by washing withRPMI-1640 medium containing 10,000 IU^−1^ sodium heparin, 100,000 IU^−1^ penicillin, and 100 mg L^−1^streptomycin twice. The mixture was then resuspended in the RPMI–1640 medium containing 3% (v/v) of pooled tilapia serum, 1,00,000 IU^−1^ penicillin, 100 mg L^−1^ streptomycin, and 4 mM L-glutamine (Biochrom AG, Berlin, Germany). The trypan blue exclusion method was used to enumerate the number of viable cells and by utilizing the culture medium; it was adjusted to 4 × 10^7^ cells [33]. 

### 2.7. Reactive Oxygen Species Production

The leukocytes of peripheral blood (1 × 10^6^ cells/well) weresubjected to incubation at 28 °C for about 2 h in the culture medium of 175 µL containing NBT (1 g L^−1^) of 25 µL for measuring the respiratory burst activity intracellularly with the slightly modified method [34]. The supernatant was discarded, and 100% methanol was added to the cells. After 5 min, the wells were washed with 70% methanol (125 µL). Anovernight air dry was performed to fix the cells. The formazan (the NBT in reduced form) was mixed to dissolve by adding 125 µL of KOH (2N) and 150 µL of Dimethyl sulfoxide (DMSO) in each well. The OD values were read at 650 nm in a microplate reader. 

### 2.8. Reactive Nitrogen Species Production

The leukocytes from the peripheral blood release nitric oxide (NO) into the medium, which is measured by Griess reagent [34]. The produced NO convertsto its stable nitrate form rapidly. The colorimetric method wasused to measure the presence of nitrite in the supernatant of culture by the adding Griess reagent to convert the nitrite into a pink color. The culturing of leukocytes involves the culture medium (175 µL) beingmoistened for 96 h at 28 °C by copper sulphate solution (1%). The culture was collected for about 50 µL followed by transferal into the microtiter plate separately. Griess reagent (50 µL), with a composition of (1% sulphanilamide, 0.1% N-naphthul-ethylendiamine, 2.5% phosphoric acid), was then added to the supernatant of the each well and then incubated for 10 min. Finally, the concentration of NO_2_ was observed by using the standard curve obtained from the NaNO_2_ concentration graded series of the culture medium. 

### 2.9. Disease Resistance

Another group of fish was used to determine the LD_50_ value of pathogenic organism, *E. tarda*, before using it for the laboratory trial and found to be 1.2 × 10^8^ cfu/fish. The broth of brain heart infusion was used to grow *E. tarda* at a temperature of 28 °C for about 24 h. Centrifugation of broth was done at 3000× *g* for 10 min. The supernatant was removed, and the obtained pellet was resuspended in PBS, which wassterile, with a pH of 7.4. The MECA was administered to the fish followed by intraperitoneal injection of *E. tarda* in the presence of PBS, at a range of 1.2 × 10^8^ cfu/mL (0.1 mL). The OD value for absorbance was adjusted to 600 nm = 1.2 for the suspension. The values for OD were calculated precedingly as the number of bacteria in 1 mL of suspension as 1.2 × 10^8^ cfu/mL. The resistance of fish against *E. tarda* was analyzed by using 10 fishes from the tank as investigational and control groups. Until 14 days, the mortality was observed. The mortality data, which was recorded, was then utilized for finding the relative percentage survival (RPS) calculated as follows [35]; RPS = (*n* − 1) [No. of Surviving fishes after challenge/No. of fishes dead due to bacterial injection] × 100. 

### 2.10. Statistical Analysis

The statistical analysis of the obtained data was performed by SPSS (Ver. 21). Results of the immunological assays except the disease resistance test were presented as mean (±standard error) for six fish per treatment group. Significant differences between groups were determined by one-way analysis of variance (ANOVA) and Duncan’s multiple range test. Mean significant differences were exploredat the 5% probability level (*p* < 0.05). 

## 3. Results

### 3.1. Growth Response

The maximum weight gain was observed in the T2-treated group, which wastreated with 75 mg/kg when compared with thecontrol (Table 1). As shown in Table 1, the results of the SGR and FCR in this study were in the optimum level. The fishes were treated with MECA at different levels with significantly (*p* < 0.05) different values between the specific and non-specific immune responses for the 7th, 14th, 21st and 28th day post treatment. 

### 3.2. Immunological Response 

#### 3.2.1. Neutrophil Activity

**Figure 1:**
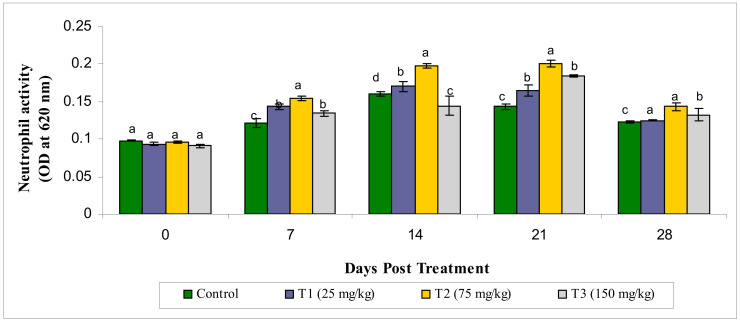
Neutrophil activity (OD at 620 nm) under different treatments administered with various levels of methanol extract of *Chaetomorpha antennina* in *Labeo rohita* during post treatment days (values are mean ± SE). ^a,b,c,d^ Mean values with different superscripts considered as statistically different (*p* < 0.05); *n* = 6 for each treatment group.

The neutrophil activity (NBT activity at OD 620 nm) of the treatment groups wasfound to have statistically significantly different values (*p* < 0.05), in comparison with the control groups and the highest value was seen in the T2 group on the 14th and 21st day post treatment. The activity of neutrophil was elevated from 7 to 14 days post treatment (Figure 1). 

#### 3.2.2. Lysozyme Activity

**Figure 2:**
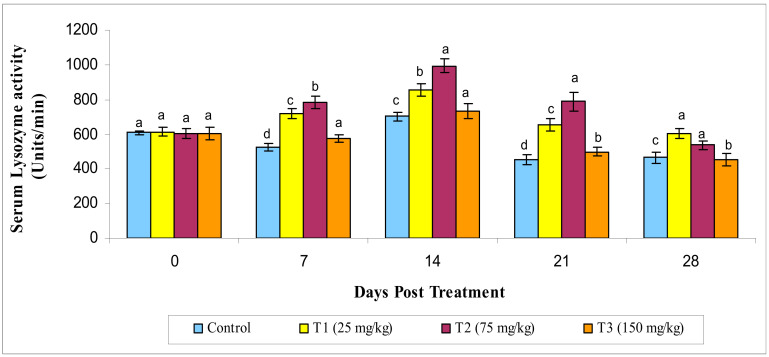
Serum lysozyme activity under different treatments administered with various levels of methanol extract of *Chaetomorpha antennina* in *Labeo rohita* during post treatment days (values are mean ± SE). ^a,b,c,d^ Mean values with different superscripts considered as statistically different (*p* < 0.05); *n* = 6 for each treatment group.

The lysozyme activity of the serum in the MECA-treated fish began to rise significantly (*p* < 0.05) from day 7 to day 21 of administration. The lysozyme activity was seen to be higher in the T2 group on the 28th day and was not significant (*p* ≤ 0.05) with the controls (Figure 2). 

#### 3.2.3. Myeloperoxidase Activity

**Figure 3:**
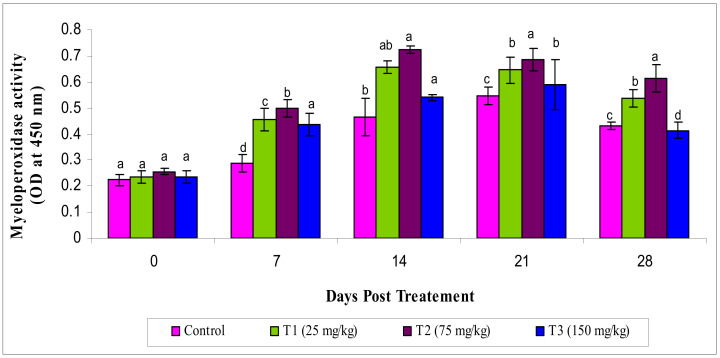
Serum myeloperoxidase activity under different treatments administered with various levels of methanol extract of *Chaetomorpha antennina* in *Labeo rohita* during post treatment days (values are mean ± SE). ^a,b,c,d^ Mean values with different superscripts considered as statistically different (*p* < 0.05); *n* = 6 for each treatment group.

The activity of MPO varied significantly (*p* < 0.05) in the experimental groups, showing an increased level of MPO in the T2 group than others (Figure 3). 

#### 3.2.4. Ceruloplasmin Activity 

**Figure 4:**
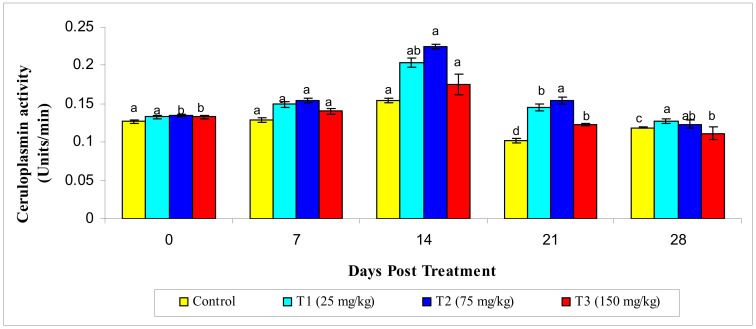
Ceruloplasmin activity under different treatments administered with various levels of methanol extract of *Chaetomorpha antennina* in *Labeo rohita* during post treatment days (values are mean ± SE). ^a,b,c,d^ Mean values with different superscripts considered as statistically different (*p* < 0.05); *n* = 6 for each treatment group.

A significantly increased value of ceruloplasmin activity (*p* ≤ 0.05) was observed in the T2 group on the 14th day post treatment compared to theother treatment groups (Figure 4). 

#### 3.2.5. Antiprotease Activity

**Figure 5:**
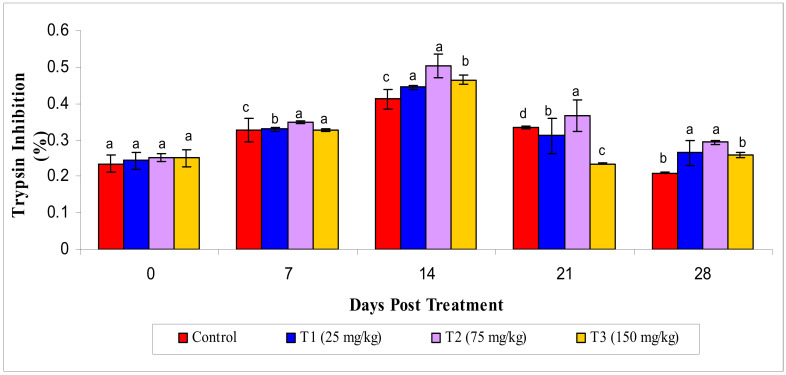
Serum antiprotease activity under different treatments administered with various levels of methanol extract of *Chaetomorpha antennina* in *Labeo rohita* during post treatment days (values are mean ± SE). ^a,b,c,d^ Mean values with different superscripts considered as statistically different (*p* < 0.05); *n* = 6 for each treatment group.

Similarly, results showed that the highest level of antiprotease activity, such as trypsin inhibition (*p* < 0.05), in the T2 group at the 14th day of the experiment compared to theother groups; however, this decreased on the 21st and 28th day of the experiment (Figure 5). 

#### 3.2.6. Reactive Oxygen Species and Reactive Oxygen Species 

**Figure 6:**
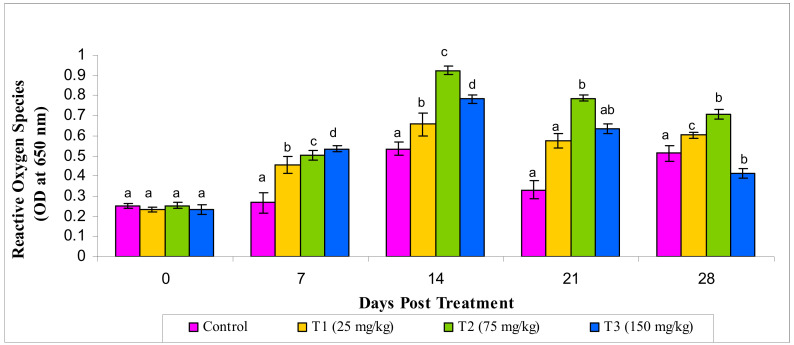
Effect of methanol extract of *Chaetomorpha antennina* on the Reactive Oxygen Species production by peripheral blood leukocytes in *Labeo rohita* (values are mean ± SE). ^a,b,c,d^ Mean values with different superscripts considered as statistically different(*p* < 0.05); *n* = 6 for each treatment group.

**Figure 7:**
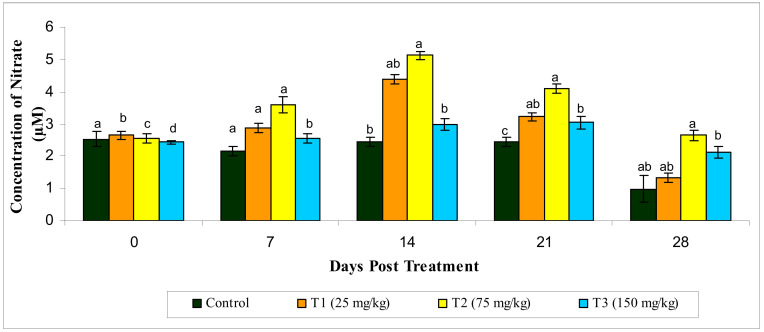
Effect of methanol extract of *Chaetomorpha antennina* on the Reactive Nitrogen Species production by peripheral blood leukocytes in *Labeo rohita* (values are mean ± SE). ^a,b,c,d^ Mean values with different superscripts considered as statistically different (*p* < 0.05); *n* = 6 for each treatment group.

Likewise, significantly increased ROS and RNS production (*p* < 0.05) was observed in the MECA-treated groups compared to the control. The highest ROS and RNS production were seen in the T2 group compared to the other treatments (Figure 6 and Figure 7). 

#### 3.2.7. Challenge Study

**Figure 8:**
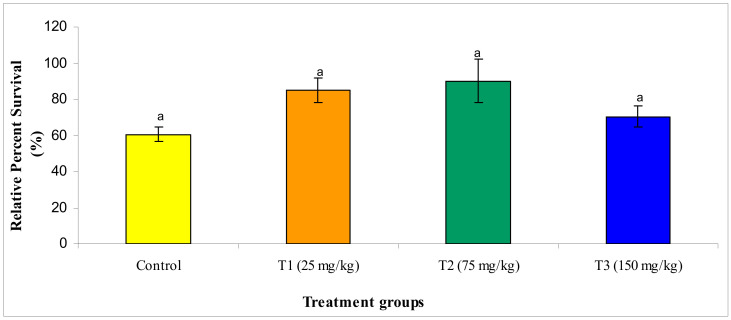
Effect of methanol extract of *Chaetomorpha antennina* administered on the relative percent survival in *Labeo rohita* challenged with virulent *Edwardsiella tarda* (mean ± SE). ^a^ Mean values with different superscripts considered as statistically different (*p* < 0.05); *n* = 6 for each treatment group.

**Figure 9:**
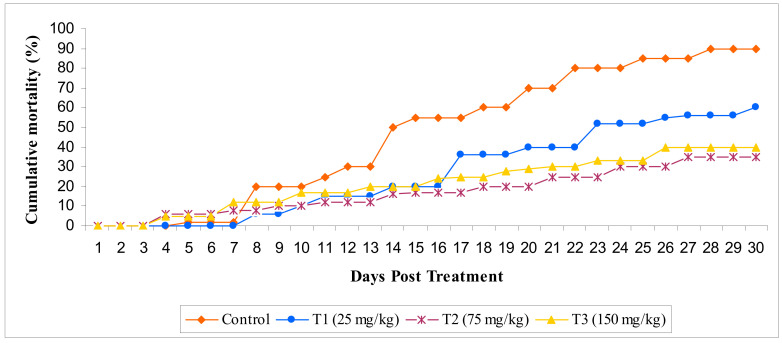
Effect of methanol extract of *Chaetomorpha antennina* administered on the cumulative mortality in *Labeo rohita* challenged with virulent *Edwardsiella tarda.*

Post challenge of fish with *E. darta*, the record of mortality was observed for 30 days. Up to 24 h, the observed mortality of fish was found to be nil. The set of fish administered with different concentrations of MECA showed an increased survival percentage, which is statistically significant when evaluated with the control. The maximum survival (%) was seen in the T2 group administered with 75 mg/kg of MECA (Figure 8 and Figure 9). 

## 4. Discussion

Special care must be taken to control opportunistic fish pathogens, which tend to cause diseases in culture organisms when they are exposed to suboptimal culture conditions during the intensification of aquaculture. Although, prophalytic treatments carried out using antibiotics have shown some success in controlling such diseases along with their negative impacts [36]. So far, there is no effective vaccine against fish pathogen in Indian conditions. To date, disease control totally relies on sound husbandry practices, good water quality, and most importantly well-balanced nutritional feed [37,38]. Our present results of conferred exposure to MECA at 75 mg/kg shows a significant specific growth rate, feed conversion rate, and immune response and disease resistance against *E. tarda*. 

The use of natural immunostimulants provides an elevated level of non-specific immunity to control disease outburst in commercial fisheries [39]. Thus, the current study starts up with the immuno-stimulatory activity of i.p injected MECA. MECA administration showed enhanced immune activities in neutrophil, serum lysozyme, serum MPO, ceruloplasmin, antiprotease, and cellular synthesis of ROS and RNS along with increased disease resistance in *L. rohita.* In the earlier study, methanol extracts of herbs like *O. sanctum, W. somnifera*, and *Myristica fragrans* were shown to have a significant effect in increasing immunogenic parameters like phagocytosis, serum activity in bactericides, ratio of albumin–globulin (A/G), and leukocratic against *Vibrio harveyi* in juvenile grouper of *Epinephelus tauvina* larvi culture [40]. Another study used methanolic extracts from five different plants, i.e., *C. dactylon*, *Aegle marmelos*, *T. cordifolia*, *P. kurooa*, and *E. alba*, in a shrimp diet preparation to reduce the white spot syndrome virus [41]. At various concentrations of the diet, such as 100, 200, 400, and 800 mg/kg, the influence was positive and significant, with a much better survival (74%) rate and reduction in the viral load. Further, an excellent execution of hematological, biochemical, as well as immunological parameters wasobserved in a diet fused with immunostimulant in shrimps [40]. In this study, MECA administration showed elevated levels of immune cells, growth response, as well as disease resistance in *L. rohita*. 

Superoxide anions are considered for their respiratory rupture property in phagocytosis of the microbes of fishes [42]. A similar study was carried out on *L. rohita* fish that were fed with a diet fused with *Achyranthus* and *Allium sativum*. This resulted in increased production of superoxide anion [43]. During the trial period, the observed fishes were injected with MECA and showed significantly increased respiratory burst activity than the control group. 

The significant defense molecule in the innate immunity is thelysozyme enzyme as it helps in arbitrating protection towards microbial intruders by splitting the linkages between the β (1→4) linking N-acetyl muramic acid and N-acetyl glucosamine in Gram-positive bacteria’s cell walls. This helps in thwarting them from the invaded host tissue [44]. In case of Gram-negative bacteria, the enzymes becomeeffectual, causing other enzymes to interrupt the outer cell wall. This results in the bacterial peptidoglycan inner layer being exposed [45]. The egg white of some avian species consists of antibacterial enzyme in the form of goose-type (g-) lysozyme. Even mammals and fishes genes are sequenced to check for homozygosity [46]. In our study, the lysozyme activity was well enhanced in MECA-treated groups. Similarly, studies showed that increased lysozyme activity is seen on day 20, 25, and 30 post feeding in Jian carp *Pseudosciaen acrocea*, a large yellow croaker [47] with *Astragalus root* (*Radix astraglinseuheydasari*) formulation asper the traditional Chinese medicine ratio of 5:1 (w/w). According to Yin et al. [48], experiments with *Oreochromis niloticus* showed *A. radix* influences the immune system positively by acting as a booster. In another study, *L. rohita* fed with the seed of *Achyranthesaspera* (0.5%) showed increased lysozyme activity [49]. Further, *Oncorhynchus mykiss* provided with *Dunaliellasalina,* a marine algae, at 100 and 200 mg/kg for a period of 9 weeks showed increased serum lysozyme activity [50]. 

Macrophage and neutrophil cells are oxidized by MPO upon entry by incursion in the fish body [49]. In the current study, MPO activity was enhanced in the both 25 and 150 mg kg^−1^ MECA experimental groups, whereas the maximum MPO activity was observed in the previous group. Comparably, there is a raise in MPO activity in oral administration of *O. mykiss* [51]. Acute phase responses are found to be non-specific in nature as it offers preliminary defense towards pathogens by raising the concentration of the plasma in various proteins involved in the acute phase responses like ceruloplasmin. The function of ceruloplasmin differs based on its activity like preventing the spread of infectious agents, revamping tissue damage, invading pathogens, and other probable microbes [44]. According to Sahu et al. [42], a significant positive correlation is found along with an elevated correlation amongst the parameters of the immune response, when analyzed in the full-sib rohu families with continued existence challenge with *Aeromoniasis*. This studies supportsthe selection of *E. tarda*resistance as a marker trait for ceruloplasmin. 

Fish plasma encloses numerous antiproteases, mainly a1 anti-protease, a2 antiplasmin, and a2 macroglobulin. These antiproteases play a crucial part in limiting the invading bacteria’s and promote invivo development [52]. In various families of rohu, a varied range of proteins and antiproteases are observed in the serum along with an unconstructive correlation with *Edwardsiellosis* [53]. Whereas in our current study, trypsin’s inhibition property was found to be increased in line with the susceptibility when compared with the resistant line, although there wasno significant difference. We also observed an optimum range of antiprotease levels in the MECA-treated groups. Further, the oxidation of certain molecules can be restrained by antioxidant molecules. Oxidation is a chemical reaction that generates free radicals, which causes chain reactions, resulting in cellular damage. Thisinhibition is responsible for reducing ROS production in metabolism. To date, research on certain algae species has provided substantial compounds with an antioxidant nature that maybe used by the industries in the field of nutraceutics and pharmaceutics [54]. Currently, varying classes of algaethat produce antioxidant compounds like carotenoids (zeaxanthin, betacarotene, lutein, fucoxanthin, and antheraxanthin), phenolics (stypodiol, isoepitaondiol, and taondiol), phycobilins (phycoerythrin), and sulfated polysaccharides (laminaran and sulfatedgalactans) have beentested, screened, and isolated [55,56,57]. 

The production of RNS was drastically increased in *Epinephelus bruneus* when given a diet rich in *Coriolus versicolor* extract for 30 days at the concentrations of 0.1% and 1.0% [58]. Additionally, in *Olive flounder* and *Paralichythy solivaceus*, fishes fed with a diet of 0.1% and 1.0% *Chaga* mushroom plus the extract of *Inonotus obliquus* for 1–4 weeks were shown to have substantially increased production of RNI [59]. The acidic polysaccharides obtained from *Ulva rigida* were accounted to provoke the rise of various chemokines’ expression two-fold by increasing the production of nitrite by macrophages [60]. In the current investigation, every dose of MECA with 25 and 75 mg/kg showed increased production of ROS and RNS in the peripheral blood by leucocytes. In various species of fish like Chinook, eel, tilapia, carp, salmon, and flounder, the disease Edwardsiellosis is caused by *E. tarda* bacterium. As a result, the infected fish showed signs of pigment loss, septicemia, enlarged kidney, lesions, necrotic abscesses, and abscesses on internal organs [61,62]. 

In our experiment, after infection, the fishes with *E. tarda* showed reduced mortality when evaluated against the control. Park and Jeong [63] stated that the protein-bound polysaccharides from *Coriolus versicolor* were injected through i.p in *O. niloticus* against *E. tarda* resulted in decreased mortality rates. In this study, MECA showed a significant reduction in the mortality rate of *L. rohita* against *E. tarda*. The screening of antimicrobial activities with several algae species like *Chnoosporabic analiculata*, *Ulva fasciata, Cheilosporum spectabile, Bryopsisplumosa, Grateloupia filicina, C. antennina, Hypneapannosa, Centroceras clavulatum, Portieria hornemannii, Gracilaria corticata, Sargassum wightii, A. orientalis, Stocheospermum marginatum*, and *Padinate trastromatica* was performed. Research on dry algal species showed a decreased level of bioactive compounds. Further, the extraction of antimicrobial compound from fresh algae amongst all other solvents was found to be effective for methanol: toluene (3:1) due to its lipophilic nature retainment for an extensive sort of temperatures (30–60 °C). Additionally, when compared to Gram-positive bacteria, these algae were shown to be more vigorous towards Gram (-) bacteria. Particularly, *Acrosiphoniaorientalis* showed an antibacterial nature against 70% of the microorganisms analyzed. *Stocheospermum marginatum* exhibited antimicrobial activity for *K. pneumoniae*, while *Gracilaria corticata* extract showed higher bactericidal activity against *P. mirabilis* [64]. The persuasive algal species, such as *Halimedaopuntia* (Order: Bryopsidales) and *Sarconemafiliforme* (Order: Gigartinales), has substantial antimicrobial, antiplasmid, and cytotoxic activity. Under the antimicrobial bioassay, *Halimeda* extract of algae species showed extensive activity when evaluated with commercially available antibiotics towards *S. aureus*.Similarly, the extracts of *Sarconema* can be regarded as an antifungal agentwith a potent growth inhibition action towards *C. albicans* [65]. 

## 5. Conclusions

To ease the management at the coastal level, the health of the fishes can be easily modulated by the use of immuno-stimulants like *C. antennina*. Among immunostimulants, the MECA through i.p treatment can reinstate the use of antibiotics and further augment the production of aquaculture. In this study, we accomplished the therapeutic prospective of *C. antennina* to improve specific as well as non-specific immunity in fishes, evidently by reducingthe mortality of *L. rohita* against *E. tarda* infection. The findings of the current study suggest a novel insight into the immunostimulation capacity of the methanolic extract after incorporating it with algae to avert bacterial disease, with the possibility of augmenting fish welfare. Consequently, we recommend including the extract till 75 mg kg^−1^ of *C. antennina* for sustainable aquaculture. Thus, the current study is amilestone for eco-safe and integrated nutritional aquaculture practices for healthy disease-resistant fish farming. 

## Figures and Tables

**Table 1:** Effect of methanol extract of *Chaetomorpha antennina* on the growth response in *Labeo rohita*.

Parameters	Control (0 mg/kg)	T1 (25 mg/kg)	T2 (75 mg/kg)	T3 (150 mg/kg)
Initial Weight (g)	50.51 ± 0.95	50.88 ± 1.48	50.61 ± 1.03	50.94 ± 2.20
Final Weight (g)	73.69 ^a^ ± 6.39	81.2 ^a^ ± 8.15	90.29 ^a^ ± 3.96	86.07 ^ac^ ± 4.81
Weight Gain	22.83 ^a^ ± 0.29	30.32 ^ab^ ± 0.30	39.67 ^a^ ± 0.19	35.13 ^b^ ± 0.21
Specific Growth rate	1.34 ^a^ ± 0.24	1.86 ^ab^ ± 0.29	1.87 ^b^ ± 0.25	2.07 ^b^ ± 0.4
Feed conversion rate	0.60 ^a^ ± 0.062	0.51 ^c^ ± 0.132	0.43 ^c^ ± 0.076	0.39 ^c^ ± 0.066

Values are expressed as mean ± SD (*n* = 12). ^a,b,c^ Means in the same row with different superscripts differ (*p* < 0.05); *n* = 6 for each treatment group.
